# Affective Scaffolds, Expressive Arts, and Cognition

**DOI:** 10.3389/fpsyg.2016.00359

**Published:** 2016-03-17

**Authors:** Michelle Maiese

**Affiliations:** Department of Philosophy, Emmanuel CollegeBoston, MA, USA

**Keywords:** scaffolding, extended mind, emotion, expressive arts, social cognition, therapy, conflict resolution

## Abstract

Some theorists have argued that elements of the surrounding world play a crucial role in sustaining and amplifying both cognition and emotion. Such insights raise an interesting question about the relationship between cognitive and affective scaffolding: in addition to enabling the realization of specific affective states, can an affective niche also enable the realization of certain cognitive capacities? In order to gain a better understanding of this relationship between affective niches and cognition, I will examine the use of expressive arts in the context of psychotherapy and peacebuilding. In these settings, environmental resources and interpersonal scaffolds not only evoke emotion and encourage the adoption of particular *bodily affective styles*, but also support the development of capacities for self-awareness and interpersonal understanding. These affective scaffolds play a crucial role in therapy and peacebuilding, in fact, insofar as they facilitate the development of self-knowledge, enhance capacities associated with social cognition, and build positive rapport and trust among participants. I will argue that this is because affectivity is linked to the way that subjects *frame* and attend to their surroundings. Insofar as the regulation and modification of emotion goes hand in hand with opening up new interpretive frames and establishing new *habits of mind*, the creation of an affective niche can contribute significantly to various modes of cognition.

## Introduction

Sterelny (2010) has maintained that mind and cognition are “environmentally supported” and highlights the way in which cognitive agents often engineer their environment so as to sustain and amplify their cognitive abilities. Drawing on this work, Colombetti and Krueger (2014) argue that the environment scaffolds not just the mind’s cognitive capabilities, but also its affective ones. They point to various ways in which agents engineer their affective environments and “in doing so let these environments influence their affective states in an ongoing way” (Colombetti and Krueger, 2014, p. 4). However, the relationship between (i) the claim that cognition is environmentally supported, and (ii) the claim that affect is environmentally supported, require further exploration. Given that affect can influence cognition, the issue arises of whether, when, and how cognition is indirectly supported by the environment insofar as (a) the environment scaffolds affect and (b) affect shapes cognition. In addition to enabling the realization of specific affective states, can affective scaffolding also provide support for certain cognitive capacities?

In order to gain a better understanding of the relationship between affective scaffolds and cognition, I will examine the use of expressive arts in the context of psychotherapy and peacebuilding. In these settings, environmental resources and interpersonal scaffolds not only evoke emotion and encourage the adoption of particular “bodily affective styles,” but also support the development of capacities for self-awareness and interpersonal understanding. These affective scaffolds play a crucial role in therapy and peacebuilding, in fact, insofar as they facilitate the development of self-knowledge, enhance capacities associated with social cognition, and build positive rapport and trust among participants. By virtue of coming together in an affective niche, participants can “work through” their feelings and heighten their understanding of themselves and others. I will argue that this is because affectivity is linked to the way that subjects *frame* and attend to their surroundings. Insofar as the regulation and modification of emotion goes hand in hand with opening up new interpretive frames and establishing new *habits of mind*, the creation of an affective niche can contribute significantly to various modes of cognition.

## Scaffolding for the Mind

Sterelny (2010, p. 466) emphasizes how “many animals intervene in their environment, shaping it in ways that improve the adaptive fit between the agent and the world.” According to his proposed niche construction model, agents adapt to their environments, but also adapt their environments to them. For example, animals construct nests, burrows, and dams; and human agents alter the informational character of their environment with the assistance of various tools. The basic idea is that environmental resources support and amplify cognitive capacities: by modifying their environment, agents are able to “alter the informational character of their environment in ways that make crucial features more salient” (Sterelny, 2010, p. 470). For example, ants lay scent trails between their nest and food source, and a hawk chooses a roost that maximizes its view of its hunting territory. This has cross-generational effects in the sense that it reshapes the developmental environment of the next generation. Once the beaver has built a dam, this changes the environment where it lives, which in turn affects the behavior of both the beaver and its offspring. As children develop into mature members of a community, environmental resources structure and amplify their cognitive processes. These “cumulatively provided tools for thinking” might be viewed as cognitive technologies afforded by a culture and include language, arithmetical notation, maps, road signs, and diagrams.

While the scaffolded mind hypothesis is closely related to the extended mind hypothesis presented by Clark and Chalmers (1998), the two are not causally and explanatorily equivalent. For one thing, the scaffolded mind thesis sidesteps the issue of whether the environment comes to be a *constitutive* part of cognition. Because it makes the weaker claim that environmental resources play a crucial role in *causally supporting* cognitive processes, the scaffolded mind thesis is able to avoid many of the objections that have been raised against the extended mind thesis. In addition, the niche-constructivist framework that Sterelny (2010) prefers is more general than the extended mind framework. He believes that environmental supports vary in at least three dimensions, each of which corresponds to a functional relationship between a resource and an agent and is a matter of degree. These dimensions are trust, individualism /entrenchment, and collectivity. When we focus on highly trusted, individualized and entrenched, single-user resources, the extended mind framework may very well be appropriate. However, this is just one of many ways in which cognition can be environmentally scaffolded.

Colombetti and Krueger (2014) aim to extend Sterelny’s (2010) niche-constructivist framework and to examine how environmental resources also support and amplify human affective capabilities. In their view, affectivity is not simply a matter of passively undergoing bodily and experiential changes, but also involves actively modifying one’s environment in order to sustain, amplify, dampen, or alter one’s affective life. What these authors call “affective niches” are “instances of organism-environment couplings (mutual influences) that enable the realization of specific affective states” (Colombetti and Krueger, 2014, p. 4). While this active manipulation sometimes is the result of conscious intention, in other instances it is just a part of one’s habitual way of dealing with the world. Some of the props and strategies used to construct an affective niche include written materials, tools for expression, bodily adornments, and works of art.

There are, indeed, a range of examples of how agents engineer their affective environments so as to influence their affective states in an ongoing way. As Colombetti and Krueger (2014) note, people rely on portable technologies for listening to music in order to feel more energetic and enthusiastic; they wear brightly colored clothing to evoke a particular mood; they look to cinemas, concert halls, and art galleries to keep themselves interested and stimulated; they use musical instruments to express and explore a variety of feelings; and they spend time with family and friends because doing so evokes certain feelings and allows them to adopt a particular “bodily affective style,” including distinctive ways of speaking, gesturing, and moving (Colombetti and Krueger, 2014, p. 7). Of course, not just any environmental resource counts as an affective scaffold. It must involve the elements of “trust” and “entrenchment” that Colombetti and Krueger (2014) cite, drawing from Sterelny’s (2010) work. Such environmental resources must be trusted, to a significant extent, to have a particular impact on one’s affective state, and also be integrated, to a substantial degree, into one’s bodily activities and daily routine. Within the context of interpersonal relationships, other people and joint activities play a central role in shaping one’s affective life. Colombetti and Krueger (2014) describe how, “by adopting particular styles—which may transform and adapt over time as other members, each with their own style, enter or leave the group and change its dynamic,” social interactors play an active role in shaping how their relationships function as scaffolding for affectivity (Colombetti and Krueger, 2014, p. 14). By emphasizing how subjects actively manipulate the material and social world for the purposes of regulating their affective condition, these authors move us away from the tendency of mainstream affective science to provide an internalist explanation of how emotional states occur (e.g., via affect programs or cognitive appraisals). In their view, the surrounding world is not simply part of the causal background, but rather plays a central causal role in the evocation and expression of various affective states.

I endorse Colombetti and Krueger’s (2014) claim that there are numerous ways in which our affective states are environmentally scaffolded by items of material culture, other people, and their interplay. However, these authors’ discussion of Sterelny’s (2010) work raises some interesting questions about the relation between affective niches and cognition that they do not yet explore. When a musician uses her instrument to explore her feelings, for example, can she also thereby come to understand herself and her surroundings in new ways? And in cases where interpersonal relationships function as affective scaffoldings, is the capacity for social cognition sometimes altered along with a subject’s affective condition? What I hope to show is that affective scaffolding often functions, simultaneously, as cognitive scaffolding. Because affective niches can support the development of particular cognitive capacities, there are numerous instances in which the environment scaffolds a subject’s cognitive abilities *by virtue* of scaffolding her affective states. To support this claim, I will examine how expressive arts can be used in psychotherapy and peacebuilding settings to evoke affective responses and, at the same time, enhance participants’ self-understandings and capacities for social cognition.

## Affectivity as a Scaffold for Cognition

Before proceeding, I should say a bit more about the distinction I am drawing between affectivity and cognition. Affective states, as I understand them, include background “existential orientations” (Ratcliffe, 2005), moods, and specifically directed emotions such as fear or anger. These modes of consciousness all involve feelings about what matters (Baier, 2004) or “feelings of subjective import” (Helm, 2001, p. 199). One might say that affective states are ways in which an individual *cares* about objects, events, states, of affairs, other people, her own life, etc. Like Colombetti and Krueger (2014), I maintain that affective states typically involve a range of components (Scherer, 2009), including bodily processes, expressions, action readiness, appraisals, and feelings; and also that they are *enactive*, i.e., they are ways of engaging with and making sense of one’s surroundings. The basic idea is that feelings of caring help us to make sense of the objects and situations we encounter and to apprehend our surroundings as an arena of possible projects and goals. Insofar as affective states involve an element of appraisal, and as the examples discussed in this paper will further reveal, there is no sharp divide between affectivity and cognition. Still, I do not wish to adopt a cognitivist account according to which affective states simply are a form of judgment. In my view, the sort of appraisal associated with affective states is pre-reflective, implicit, non-algorithmic, and fully bound up with bodily feeling (Maiese, 2011, 2014).

Alongside these *feelings of caring*, there is also the capacity for various modes of thought. When I speak of ‘cognitive capacities,’ what I have in mind is cognition in the traditional sense, as outlined by Sterelny (2010): the abilities associated with various modes of thought, judgment, problem-solving, and intelligent action. The particular modes of cognition I discuss in this paper are social cognition and the meta-cognition associated with self-awareness. My focus, then, concerns whether the evocation of particular feelings of caring can “alter the informational character” of one’s environment (Sterelny, 2010, p. 470) and thereby enhance or assist various modes of thought and problem-solving. I believe that the discussion I present here not only shows how affective scaffolds can support relatively sophisticated cognitive abilities, but also lends support to an enactive account of affectivity.

But why think that affective scaffolds make a significant contribution not only to the emotional dimension of people’s lives, but also to the cognitive dimension? This is because central to cognition is the ability to gauge significance and home in on relevant features of one’s surroundings. As subjects navigate their way through the world, obviously they do not sequentially process all of the cognitive and practical information that is potentially available to them, but instead almost always home in on certain very specific things rather than others. Although they tend not to notice it, some degree of attention focusing takes place in all of their interactions with their surroundings, including sensory perception, decision-making, and more abstract thought processes.

This ability to home in on salient features of our environment and make a cut from the stream of information is what cognitive scientists call ‘framing.’ If intentional directedness did not involve an underlying process of *framing* and selective attention, then agents in the world would be faced with a potentially endless array of possible cognitive and interpretive options, and presumably would merely shut down from information overload. This is because it is by virtue of framing that we are able to find definite points, lines, and contours of salience in the complex world around us, and thereby able to orient ourselves in that world. Some aspects of our surroundings and situation come to the foreground, while others fade to the background. The way in which we frame an object, situation, or event, which essentially involves highlighting some considerations while overlooking others, carves out the “starting points” for thought and other cognitive processes.

Now, it is true that concepts help us to organize complex information into coherent categories and thereby get our cognitive and practical encounters with the world ready for judgment, inference, and self-conscious deliberative intentions. However, most subjects also have an immediate, pre-theoretical, non-intellectual understanding of where to direct their attention in a given context. Detection of which aspects of our surroundings are relevant typically occurs outside of reflective self-awareness, is non-algorithmic, and involves bodily attunement and feelings of subjective import. Bodily affectivity permeates our interpretations and patterns of attention and thereby enables us to make sense of the world. Drawing from the work of Husserl, Ratcliffe (2005) uses the notion of a ‘horizon’ to capture the sense in which the feeling body serves as a framework through which world-experience and interpretation are structured. What he calls an ‘existential orientation’ is the subject’s general sense of her relationship to the world, which is constituted by bodily feelings and can be understood as a space of possibility that determines the various ways in which things can be experienced. Along similar lines, Thompson (2007, p. 374) maintains that affect operates as the “allure” of consciousness, and implies a “dynamic gestalt or figure-ground structure” whereby some objects emerge into affective prominence, while others become unnoticeable. This means that a subject perceives and evaluates her world through a corporeity that is always already affectively nuanced (Colombetti, 2007, p. 542).

Elsewhere, I have characterized this process of discriminating, filtering, and selecting information in terms of “affective framing.” Just as a conceptual frame is a cognitive shortcut that people rely on in order to categorize features of their surroundings, an affective frame operates as a feeling-driven shortcut whose interpretive focus is targeted and contoured by an individual’s embodied desires and cares. Affective framing habits, developed over time, yield a *pre-reflective*, fine-grained contouring of a subject’s surroundings, so that she immediately can target and focus her attention. As a result, subjects interpret persons, objects, facts, states of affairs, and situations in terms of what they care about. Which information is relevant to a subject’s thoughts, for example, is partly a function of her desires, concerns, and overall perspective, so that the bodily feelings of affective framing function as a structure-giving background to all experience and a presupposed context for all intellectual, practical, and social activity. This *caring-contoured map* of affective frames also plays an important role in determining which information in working memory will be held onto, which will fade out, and which will be called into conscious attention when needed. Affectivity and bodily feeling thereby bias the competition for processing resources in favor of information that subjects *feel* is important. Such framing determines subjects’ attentive focus, right down to the most fine-grained levels, and thereby fixes precisely which features of their surroundings become salient for them. If subjects did not rely on affective framing, then they would be faced with a potentially endless array of possible cognitive and interpretive options, and this would make the task of adapting to their environments very difficult.

Affective frames correspond to the practical, existential orientation which Ratcliffe (2005) maintains structures all experience. They also encompass what Colombetti and Krueger (2014) call a “bodily affective style,” which is an individual’s characteristic manner of comportment, including her distinctive ways of speaking, gesturing, and moving. These bodily habits play a central role in our conduct, and represent “our typical and cultivated ways of integrating and interacting with the environment” (Cuffari, 2011, p. 537). Along these lines, Sheets-Johnstone (2011, p. 160) describes how, over the course of learning to move our bodies, we forged a large number of dynamic patterns that became habitual. For example, brushing one’s teeth, tying a knot, and writing one’s name all were woven into our bodies as familiar dynamics, and came to “bear the stamp of our own qualitatively felt movement patterns, our own familiar synergies of meaningful movement” (Sheets-Johnstone, 2011, p. 160). Similarly, Colombetti (2014, p. 62) describes emotional expression as a “coordinative structure” and maintains that “adult expressions can be characterized as relatively recurrent and fixed patterns whose specific shape has been carved in development as certain structures occurred more frequently.” This includes breathing patterns, facial expressions, postures, and characteristic gestures, which together form a subject’s emotional comportment and patterns of bodily attunement.

It is important to emphasize, though, that a subjects’ “bodily affective style” and bodily habits include not just their customary emotional responses and movement repertoire, but also their characteristic patterns of attention and habits of interpretation. Over time, subjects develop a particular “style” of experiencing the world, and their associations, habits of perception, and patterns of attention are sedimented in the body. In my view, the notion of ‘affective framing’ helps to conceptualize the idea that built-up patterns of bodily attunement and response serve as a form of “operative intentionality” that encompasses a wide range of perceptual and behavioral dispositions (Summa, 2012, p. 23). These affective frames or “habits of mind” involve schemas for interacting and engaging with one’s environment, and include, for example, a tendency to notice particular features of people and events while ignoring others; to naturally trust some sources of evidence while remaining suspicious of others; to listen carefully or be easily distracted; and to pick up on subtle nuances of others’ behavior, posture, and tone of voice.

Crucially, as Colombetti and Krueger (2014) note, such patterns of attention and response are not fixed or static, but rather “loosely assembled,” dynamic, and susceptible to ongoing modification. First, a subject’s affective style or affective framing patterns can shift depending on what sort of niche or social context she inhabits. For example, someone might exhibit one sort of affective style while one is teaching and a noticeably different type of affective style when one is interacting with one’s friends. Second, a subject’s bodily affective style can change more significantly over the course of learning and socialization, and in response to life experiences. Sometimes this change is gradual and the alteration to a subject’s patterns of attention, sensitivity, and response is relatively subtle. In other cases, change is more sudden and dramatic, habits of mind reconfigure, and subjects undergo changes to bodily comportment and a pronounced “affective re-orientation.” Whether the change is gradual and subtle, or more sudden and significant, a shift in affective framing can open up new avenues for thought, engagement, and response. As a result of a shift in affective framing, subjects may become more receptive to certain kinds of information, as well as more able to appreciate the relevance and salience of factors that previously had remained obscure. In short, when a subject’s affective frames and bodily affective style shift, she develops what might be deemed “new habits of mind” that alter her characteristic ways of attending to, making sense of, and actively engaging with their surroundings. What I will argue is that these shifts in affective orientation can serve as scaffolding for new modes of thought and cognition. This is because a shift in affective framing changes not just how one perceives the world, but what someone remembers, what one attends to during practical reasoning, and what one notices about other people. Insofar as affective niches serve to evoke certain kinds of emotional states, they also have the potential to guide participants’ attention and foster particular affective framing tendencies.

In order to examine further how affective scaffolding simultaneously serves as scaffolding for cognitive processes, I turn to expressive arts as an example of one way in which subjects modify and shape their social environment so that it fits their human purposes and goals. Sterelny (2010) notes how minded creatures purposefully alter the character of their environment so as to make crucial features more salient (Sterelny, 2010, p. 470). Building on this work, I maintain that creating an environment that evokes particular kinds of affects and bodily feelings likewise can function as a way to guide subjects’ attention, allowing them to notice particular features of their surroundings while ignoring others. Within the settings of therapy and conflict resolution, material items and other people sometimes interact in the way Colombetti and Krueger (2014, p. 16) describe to construct a particular “affective niche,” one which not only allows for particular affective states to take shape and thrive, but also facilitates self-understanding, perspective-taking, and social cognition. In fact, it is reasonable to suppose that expressive arts are introduced in these settings precisely so as to engineer a particular sort of affective environment, one which can amplify various kinds of cognitive functioning. For example, one important way in which the Holocaust has been commemorated and its full implications understood is through an appeal to visual art, music, and ceremony. It appears that such activities allow people to gain insights about the dynamics of conflict and comprehend what sorts of relationships need to be fostered in the future.

I propose that the scaffolding provided by expressive arts serves to sculpt participants’ affective framing patterns, and that this amounts to the modulation of their habits of attention, engagement, and response. In some instances, the *impersonal* environment shapes affect, and non-socially acquired affective frames go on to influence and support cognition. In other instances, affective frames are socially acquired, and also enable new forms of social interaction. These new interpersonal possibilities, in turn, serve to further modulate affect, which then influences cognition, so that a complex social-affective-cognitive dynamic emerges. Indeed, as the examples I present will show, a wide range of social-affective-cognitive interactions are possible. Nonetheless, these cases all demonstrate how affective reframing can contribute significantly to various modes of cognition. This is because by evoking various emotions, expressive arts pave the way for a pronounced alteration in affective orientation and the formation of new habits of mind. The case of expressive arts therefore helps to illustrate “the transformative powers of environmental scaffolds” (Colombetti and Krueger, 2014, p. 3) with respect to both affectivity and cognition.

## Dance-Movement Therapy and Self-Understanding

DMT can include rhythmic dance, spontaneous and creative movement sequences, thematic movement improvisations, unconscious symbolic body movement, and a range of relaxation exercises (Mills and Daniluk, 2002, p. 78). Sessions typically occur in group settings, and begin with a warm-up phase in which subjects prepare their bodies for action, followed by an exploration phase in which participants experiment with various non-directive, improvised movements. The therapist may try to match the rhythmic components of the music to the energy level and movement qualities of the group. Some of the participants’ movements that seem most meaningful and emotionally significant then are selected as “themes” to be explored further during the core action phase (Ren and Xia, 2013, p. 6). This sort of semi-structured format provides a safe space for some degree of spontaneity and creative expression. In such settings, the physical layout of the space, the positioning of participants, props, music, and particular movement sequences come together to create a specific affective niche that allows participants to “work through” through emotions via movement, expressive action, and various forms of bodily engagement. In this section, I will argue that in cases where DMT is particularly effective, this regulation of affectivity via movement can contribute significantly to self-understanding. These are cases in which non-socially acquired affective frames influence and support cognition.

Insofar as free movements together with rhythm typically evoke both intense physical sensations as well as strong emotions, the DMT setting clearly qualifies as an instance of affective scaffolding. Indeed, DMT provides us with a powerful example of how of how subjects sometimes *actively* modify their environment in order to sustain, amplify, or dampen their affective lives (Colombetti and Krueger, 2014, p. 4). As movement sequences unfold, there are opportunities for increased emotional expression and the controlled, cathartic release of emotions of joy, sorrow, rage, or frustration. Specific movements not only reflect certain “inner psychic realities,” but also “call forth images moods, and memories” (Baum, 1991, p. 99). Because movement expresses affective states, articulates attitudes, and presents the subject’s personality, it can be a powerful way to tap into subjects’ affective condition. Often a certain affective “style” comes to characterize the dance therapy setting, one which fosters particular kinds of moods and emotions.

Mills and Daniluk’s (2002) description of the experience of dance therapy for adult women survivors of child sexual abuse helps to show how the exploration of emotion through movement can facilitate self-understanding. These authors conducted in-depth interviews with five women who had endured sexual abuse as children and had completed six sessions of dance therapy. Their qualitative study was intended as a phenomenological exploration into the lived experience and meaning of dance therapy for participants and examined the ways in which it seemed to them to facilitate personal growth and healing. All of the women in the study described becoming more aware of their body parts and physical sensations and more comfortable with the range of emotional sensations they experienced. These individuals believed that dance therapy helped them to learn an alternate response to the resurfacing of painful memories. Previously, they had tended to cope with discomfort by “going into their heads” and intellectualizing. Dance therapy offered them a way to bypass this defensive reaction to discomfort and kept them connected to their bodily sensations and affective states. It also offered a way to discover *bodily truths*, i.e., an awareness of how deeply their bodies felt things that had happened to them in the past. By unlocking this sort of “body memory,” the participants were able to increase their emotional awareness and more accurately identify their deeper feelings. They found that “working in the medium of movement rather than just words helped to make their emotional worlds more accessible to them” (Mills and Daniluk, 2002, p. 80). Note that this increased self-awareness is not simply a matter of gaining access to new evidence, but also involves refocusing one’s attention on relevant aspects of one’s memories, desires, perceptions, and beliefs.

In many cases, free associations in movement also can allow for the symbolic expression of material that is too painful for subjects to verbalize (Koch and Harvey, 2012, p. 382), and because they are not restricted in their use of space or the intensity of their emotional expression, many participants are able to have breakthrough cathartic experiences. Insofar as movement allows for the release and externalization of emotion in a safe setting, it has the potential to allow participants to become more aware of buried emotions or inner conflict. Indeed, many subjects described dance as a unique way to experience some sort of emotional release:

“My epiphany was a complete body experience. I started sobbing, and I ended up on the bathroom floor curled up in the fetal position screaming at my dad, screaming at my mom, that I wasn’t going to hold onto this anymore…… and my body let it go. Since then, I remember the beatings and the sexual abuse, but the pain isn’t attached to it anymore. I’m not reexperiencing the pain with every memory” (Mills and Daniluk, 2002, p. 82).

Participants also reported that so-called “authentic movement” emerged: they were able to follow the inner impulses associated with their sensations and images and let their movements unfold naturally, being true to their perceptions, sensations, and impulses. One participant said that dance therapy helped her to “open the passages to her emotions and to express herself in a more authentic way,” so that she moved more naturally and felt freer in how she expressed her feelings (Mills and Daniluk, 2002). Along similar lines, Koch and Harvey (2012, p. 377) describe how “punching out” forms an important part of some subjects’ dance sequences: subjects punch outward, and in doing so, are able to express and reconnect with their feelings of anger and aggression.

Such testimony suggests that in addition to serving as scaffolding for affectivity, dance and movement offer a way for subjects to reconnect with their feelings and become *more aware* of their inner emotional worlds, including their reactions, beliefs, behavioral tendencies, and memories. Movement statements might be understood as “kinetic explorations” (Pallaro and Fischlein-Rupp, 2002) of images and themes that often are outside conscious awareness and yet characterize the participants’ outlooks on the world (Baum, 1991, p. 100). Because bodily states, unconscious feelings, beliefs, and desires all are interconnected, enhanced experience and processing of bodily and affective states can contribute to self-awareness and overall cognitive functioning (Mills and Daniluk, 2002, p. 78). For one thing, by “grounding” subjects and helping them to “stay with” their uncomfortable sensations and emotions, dance allows them to develop more adaptive ways of coping with stress and discomfort. The hope is that once buried emotions and memories come to the surface, subjects will be in a better position to “work through” painful feelings and examine them further.

In addition, because spontaneous movement and emotional expression helps subjects to reconnect with their bodily sensations and feelings, it can promote increased bodily awareness and a stronger and more stable sense of self (Gorham, 1995, p. 363). Dance allows participants to express themselves without thinking first, and without the need for words or any sort of verbalization. As subjects move beyond their cognitive defenses and get a better grasp on how they are feeling, they also, at the same time, gain increased awareness of their thought processes and a deeper sense of how past experiences continue to impact their thoughts, beliefs, and behavior. When particular emotions “come out,” this can spark recognition of salient factors regarding a subject’s own life and situation that previously had gone unnoticed. This counts as a cognitive shift, though one that very well may occur outside of reflective awareness. Particular aspects of their thought processes and behavioral tendencies that they may not have attended to prior to the emotional episode come to the forefront. One might say that the problem space of self-understanding is shifted from the individual’s head into the space where movement sequences unfold. Insofar as bodily expressivity paves the way for meaningful insights about one’s thoughts and feelings, it can serve as scaffolding for increased self-awareness and even, perhaps, processes of *self-reflection.*

DMT also allows subjects to modify their *movement repertoire* and thereby shift their habitual patterns of engagement. Gorham (1995, pp. 363–364) rightly notes that “self states” can be observed on the surface of the body in the form of tensions, some variable and some fixed, which are continually changing. A subject’s “movement repertoire” is constituted by the range, characteristics, and frequency of these changes. One movement attribute is *tension flow*, which is defined by the bodily response to rhythmic changes in breathing, other inner stimuli, and outer stimuli. Tension flow involves not just the amount of tension localized in one place on the body, but instead “the quality of changes in tension throughout the body that cast the forms (shapes) and patterns” of the subject’s movement repertoire (Gorham, 1995, p. 364). When someone is engaged in an activity, the tension flow states increase, take on a particular bodily shape, and involve a particular quality of effort. The very same movement can have very different qualities and meanings depending on the speed or intensity with which it is executed (Colombetti, 2014, p. 119). Because subjects express their desires and concerns through movement, a shift in characteristic patterns of movement can involve a corresponding change to their affective frames and “habits of mind” that alters the way in which they make sense of themselves and their situation.

Modified affective frames can allow subjects to become more keenly aware of pertinent facts, more attuned to their own personal blind spots, and more capable of detecting oversimplifications, exaggerations, and false dichotomies in their own thought processes. *Affective reframing* therefore has the potential to shift the way that subjects make sense of their everyday struggles and allow them to become more aware, for example, of how their feelings of fear or anger have a continuing influence on their behavior. This enhanced capacity for selective attention constitutes an improvement in subjects’ problem-solving abilities insofar as it facilitates more accurate meta-cognitive judgments about their beliefs, desires, and overall state of mind. As a result of this shift in affective orientation, subjects are better able to arrive at meta-cognitive judgments of the form, “I am afraid of X,” “I am angry about Y,” and “I remember Z.” DMT thereby serves as a striking example of an affective niche that allows subjects to attend appropriately to their emotions, desires, and needs and thereby increases their overall self-awareness.

## Dance and the Creation of “We-Space”

In the previous section, I described how cognitive processes associated with self-awareness can be facilitated via non-socially acquired affective frames. However, in other instances, interpersonal interaction plays a more central role. In these cases, the social environment shapes affect in such a way that it opens up new possibilities for interpersonal interaction. These new possibilities, in turn, further re-shape affect, which influences and supports social cognition. Baum (1991, p. 101) describes how a group session might unfold: the group sits in a circle of chairs, and the leaders check with each member, both verbally and non-verbally, to gauge their mood. After an initial warm-up, the group stands and begins to move. At times, synchrony develops and the participants move together rhythmically; but in other cases, individuals take turns initiating movement sequences, and other members of the group either observe that movement or try to match the movement quality of the initiating subject. The goal is to mimic that subject’s quality of movement and to resonate with it in order to get a sense of the emotions that are being expressed (Koch and Harvey, 2012, p. 375). As the members take turns executing new movements and mirroring each other, they become more aware of feelings and states of mind associated with interactional movements and also begin to appreciate others’ perspectives (Pallaro and Fischlein-Rupp, 2002, p. 38). On the one hand, the mirroring of someone else’s movement increases one’s capacity for empathy and the ability to use one’s own body for interpersonal resonance. Group members learn how their movements affect others as well as how they react to others’ movements. Imitation and synchronization can serve as a means of inviting others into a relationship, conveying acceptance, and building trust (Colombetti, 2014, p. 200). On the other hand, subjects who have an opportunity to direct the group DMT session can gain a sense of agency and control, and the experience of being mirrored typically brings with it a sense of validation and recognition. Not surprisingly, then, moving in synchrony or coordinating movements with others can foster feelings of togetherness, trust, and positive rapport. In fact, the intense bodily resonance that arises via dance and movement sometimes might even be viewed as a matter of “kinesthetic empathy” (Baum, 1991, p. 101).

The case of DMT powerfully illustrates how interactions between various individuals within a collectively structured environment can function as affective scaffolding and enable the achievement of affective states that are inaccessible to the isolated subject. This is because the initiation of particular kinds of movements evokes emotion in oneself as well as the other participants. Both the subject who initiates the movement, as well as those who imitate or move in synchrony with her, come to experience specific kinds of affective states that depend in part on the expressions and behaviors of others. To some extent, this might be understood as a matter of emotional contagion or what some theorists have described as “automatic emotional resonance” (Hatfield et al., 1994, p. 2). When other people laugh, we are inclined to laugh too; when other people are anxious, we become anxious; and when others are at ease, this puts us at ease. Similarly, the movements and expressions of someone engaged in a DMT therapy session influence and modulate the emotions and expressions of other participants. Thus, the case of DMT shows how “members of a group may provide ongoing resources and feedback that scaffold the experience and expression of emotions unique to a certain context” (Colombetti and Krueger, 2014, p. 11).

The affective states that are scaffolded by DMT (such as trust and empathy), in turn, open up new interpersonal possibilities and facilitate the construction of what Krueger (2011, p. 644) calls “we-space”: “an emotion-rich coordinative space dynamically structured via the ongoing engagement of social agents.” A shared we-space centers upon co-regulated interaction, whereby participants continually and mutually adjust their expressive gestures, gaze patterns, vocalizations, and actions. Bodily expressions serve as “expressive scaffolding” for social cognition in the sense that they convey extensive information about individuals’ moods, emotional states, intentions, and behavioral competence. The establishment of we-space counts as a niche that transforms the informational load placed on individual agents and opens up new opportunities for thought and action. Krueger (2011, p. 653) points to various examples of interactional synchrony between infants and caregivers and describes how “the rhythmic coupling of patterns of expressive movements” serve as bodily expressive proto-conversations. From the very beginning of a child’s life, other people use expressions and gestures to invite him into some sort of communicative activity with them. For example, a caregiver makes a pointing gesture and draws the child’s attention to something she wants him to see. Infants as young as 4 months old are attuned to the eye gaze of others and use eye gaze cueing to help them process objects. In addition, joint attention helps young infants to recognize the relevance of social information, and they rely on eye contact and tone of voice to determine when information is intended for them. Likewise, within the context of dance movement therapy, participants have access to fine-grained social information over the course of dynamic, embodied engagement. During this direct, face-to-face social encounter, the movements and emotional expressions of one party orient the attention and interpretive activity of the other and they come to share in each other’s perspective and point of view.

As a result of these new interpersonal possibilities, participants’ need to rely on verbal expression is diminished. This is crucial given that subjects often find it very difficult to express their emotions and concerns in words. Movement sequences, the manipulation of space, the positioning of participants, and the choice of music serve as tools for the exploration of emotion and increased social understanding. The interactive character of this engagement alters the structure of that particular cognitive niche (the we-space), and generates new processes of shared feeling and understanding that are specific to that exchange (Krueger, 2011, p. 646). What I wish to emphasize is that this cognitive niche is also, simultaneously, an affective niche, and that both social cognition and affect depend on the scaffolding provided by the dance movement therapy setting. And this is no accident. Imitation and the coordination of movement evokes emotion and fosters empathic attunement, which in turn allows participants to understand each other. Via the alignment of movements, gestures, and vocalizations, participants are able to share intentions and construct felt contexts of sympathetic attunement (Krueger, 2011, p. 649). This strengthens their capacity for interpersonal understanding and also can allow new meanings to emerge over the course of the encounter.

I have suggested that the new interpersonal possibilities provided within the DMT setting further re-shape affect and thereby support social cognition. One way to make sense of this is in terms of the mutual modulation of the participants’ affective frames. Gallagher (2005) suggests that social cognition involves processes that occur on the level of bodily sensations, in particular kinesthesis, or sensory experience of one’s own movement. When we see someone else act in a certain way, our own kinesthetic system is activated in a way that mirrors the perceived action of the other person. Likewise, when we see someone else engage in expressive activity, our own bodily affective system is activated in a way that mirrors the expressions of the other person. Throughout the group DMT session, subjects modulate, and are being modulated and affected by, the expressions, gestures, and actions of other participants, and this mutual modulation impacts their perspective and habits of interpretation. Through imitation, synchronization of gestures, mimicking of postures, turn-taking, and other sorts of coordination, subjects may shift their point of view and begin to develop new habits of openness and responsivity. Just as a smiling face invites us to smile back, people dancing invite us to join them in that activity and share in their feelings. We involuntarily attune to them with a mimetic response, and our patterns of bodily attunement shift.

Ratcliffe (2007, p. 149) rightly maintains that these dynamics of “mutual influence” are rooted in “a distinctive kind of bodily responsiveness comprised, at least in part, of patterns of motor-readiness and an affective sensibility to gestures and expressions” (Ratcliffe, 2007, p. 158). Insofar as their affective frames (their patterns of focus and attention) are correlated and coordinated, the interactors are made interdependent and their desires and point of view are altered. Once two or more interactors become part of a coupled system, their bodily dynamics, postures, and expressions become coordinated to some extent, so that each person’s behaviors modulate the affective frames of the other participants. What is more, each subject understands the desires, thoughts, and emotions of the other participants in large part because of the impact that their expressive behavior and action have on his perspective and emotions. Sharing in one another’s feelings allows subjects to share in one another’s point of view.

In addition, because each individual’s “habits of mind” are shifted over the course of the interaction in accordance with other participants’ desires and concerns, face-to-face interaction has the potential to bring about a pronounced “affective re-orientation” that shifts the perspective of each of the parties involved. By evoking emotion and encouraging the adoption of a particular bodily affective style, group DMT can shift the way that participants *frame* their personal struggles as well as their relationships with others. Affective reframing enhances their capacity to attend to fine-grained social information, and participants become more able to detect subtle aspects of others’ bodily comportment, gesture, and expression. Again, this occurs in large part via the ‘motor resonance,’ bodily reverberation, bodily attunement, and bodily coordination that takes place in the DMT setting. By *moving together*, subjects are able to *create meaning together* and to gain new understandings that were unavailable to them as isolated subjects. Group bodily coordination thereby opens up a “shared bodily affective space” (Colombetti, 2014, p. 201) in which new opportunities for social understanding can arise. This illustrates how the provision of certain kinds of affective scaffolding can strengthen people’s capacity for social cognition. In addition, as I will discuss in the next section, this increased capacity for intersubjective bodily attunement can pave the way for more sophisticated forms of perspective-taking and joint understandings.

## Artistic Expression, Participatory Sense-Making, and Conflict Resolution

Examples from the field of conflict resolution add further support to the claim that expressive activities can open up new ways of interacting and relating and cultivate an “openness” to others’ perspectives. As Yorks and Kasl (2006, p. 52) note, expressive activities provide subjects with a “brief portal of entry” into another individual’s unique point of view. By “affording glimpses into the other’s world of experience,” (Yorks and Kasl, 2002, p. 187) and allowing participants to share their feelings, expressive activities can open up new opportunities for perspective-taking and help to establish empathic connections. In part this is because by evoking certain kinds of emotion, these expressive activities can enable subjects to experience a radical transformation in consciousness that has the potential to dislodge their habitual assumptions and to recognize aspects of their situation that previously had gone unnoticed. One might say that expressive activity helps to shift the problem space of social understanding from individual heads into the interactive space of embodied engagements (Krueger, 2011, p. 653). Such activities function by way of emotional exposure and bodily engagement, rather than conventional intellect, to shift participants’ perspectives and habits of mind. This can be crucial in conflict resolution settings, where subjects need to work together to move toward solutions, and yet may find constructive verbal dialog difficult to initiate. In fact, in cases of intractable conflict, mere persuasion or the provision of more information is unlikely to transform how subjects frame their situation. Because expressive activities offer a way to shift individuals’ “cognitive and emotive path” (Long and Brecke, 2003, p. 28) and thereby foster new understandings about identity, relationships, and ways forward (LeBaron, 2002, p. 139), they have the potential to be even *more effective* than explicit negotiation and verbal dialog in some cases.

In the previous section, I maintained that the incorporation of expressive arts can be viewed a way to engineer and exploit we-space in order to sculpt parties’ attention and foster new habits of mind. This is one way in which social interaction shapes affective framing. In addition, the use of expressive arts in conflict resolution settings can contribute to what De Jaegher and Di Paolo (2007) call ‘participatory sense-making’: “the coordination of intentional activity in interaction, whereby individual sense-making processes are affected and new domains of social sense-making can be generated that were not available to each individual on her own” (De Jaegher and Di Paolo, 2007, p. 497). There are a wide range of examples that illustrate how, through the coordination of intentional activity, the way that each individual understands a situation is mediated by the sense-making activities of the other person(s) involved in the encounter (De Jaegher and Di Paolo, 2007). Just as expressive behaviors such as smiling, crying, or blushing are ways of modulating the affective states of others, engaging in expressive activities can serve as a way to for participants to influence one another’s affective states and points of view. Affective coordination is particularly evident in situations of intricate bodily coordination, such as group dance, in cases where there is motor mimicry, matched or coordinated body positions, and complementary movements or gestures. Likewise, working together on a creative project involves bodily coordination that can modulate participants’ affective states and create a space in which new meanings can arise; and even watching someone else dance or listening to music can evoke emotion and thereby shift the way that participants view their situation. This sort of social interaction modulates affect and thereby shapes and supports cognition.

One example of participatory sense-making that De Jaegher and Di Paolo (2007) present is that of Janet, who stands in front of an open window and takes an appreciative breath of fresh air in such a way as to make sure that John notices it. In their view, this is a communicative act: Janet wants John to attend to a particular salient part of the world and notice certain things that are visible to both of them, “to engage imaginatively with certain possibilities which these things present,” and “to frame the visible world in a certain way” (De Jaegher and Di Paolo, 2007, p. 499). John understands Janet’s desires, thoughts, and emotions in large part because of the impact that her expressive behavior and action has on his perspective and patterns of attention. His affective orientation and bodily dynamics are shifted over the course of the interaction in accordance with Janet’s desires and concerns, and this shift goes a long way in helping him to understand Janet’s perspective. Similarly, during a game of charades, all of the interactors must adjust their sense-making so that it converges toward the ‘right’ gesture and the ‘right’ interpretation. The meaning of gestures is jointly constructed and transformed during the game. Here each individual’s “understanding of the other person is constituted within the perception-action loops that define the various things that [she] is doing with in or in response to others.” (Gallagher, 2008, p. 168).

Likewise, in conflict resolution settings, expressive activities can function as communicative acts whereby subjects try to adjust other participants’ “cognitive and affective take on the world” (De Jaegher and Di Paolo, 2007, p. 499). Such activities have the potential to draw parties’ attention to particular features of other individuals’ emotional states and perspectives, to highlight previously unnoticed considerations, and to make certain facts more salient. In addition, the incorporation of expressive arts may help to foster a mood of trust, promote feelings of empathy, and thereby foster a “bodily affective style” among the participants that is conducive to peacebuilding. It seems, then, that expressive arts shape affect (e.g., by fostering a mood of trust), which then opens up new possibilities for interaction; and such interactions, in turn, pave the way for affective reframing that has the potential to shift parties’ understanding of their situation.

One example of the use of expressive arts in a conflict resolution setting comes from Northern Ireland. In August of 1998, after the ceasefire had been declared, the last major bomb went off in the town of Omagh. Twenty-nine people died and many feared the collapse of the Irish peace process and a return to violence. The public responded by sending flowers and wreaths, which arrived by the 100s to fill the town streets. In response to questions about what to do with all the flowers, an artist named Carole Kane proposed that they use them to make paper. In what came to be known as Petals of Hope, people from all walks of all life and from both sides of the conflict gathered in a series of workshops. As they transformed the flowers from the wreaths and arrangements into textured paper, people talked about their experiences and where they had been when the bomb went off. Through creative activity, many people were able to acknowledge and deal with their loss and continue to move toward a peaceful future (Lederach, 2005, p. 157). I hypothesize that this creative activity served as scaffolding for constructive dialog, the “working through” of emotions, and the process of building peace. Material elements of this scaffolding included the flowers and wreaths, the tools participants used to transform them into textured paper, and the physical space in which the workshop took place. And interpersonal elements of this scaffolding included the patterns of interaction and dialog established among the participants. This joint creative activity encouraged the adoption of a certain kind of bodily affective style and mood among the participants, one characterized by collaboration and the sharing of personal experiences. Such activity likely changed not just how subjects felt about the conflict, but also, simultaneously, the way they framed their future together. Joint participation in this loosely coordinated activity thereby made a positive contribution to their capacity to share meanings and understand each other.

Another sort of expressive activity that people throughout the world rely on to prevent and manage conflict and to move toward reconciliation is *ritual*. Ceremonies and ritual customs contribute to communal harmony and individuals’ emotional well-being by giving them an outlet for their feelings of grief, anxiety, or anger. Rituals such as dancing, art, ceremonies, and holiday traditions rely heavily on sensory cues and symbolic activity as a form of communication. The smudging ceremony is one example of a peacebuilding ritual that is used in many Native American communities to address various forms of conflict. A Native Elder usually leads the ceremony and begins by lighting a mixture of cedar, sweetgrass, tobacco, and sage and fanning the burning mixture to direct the smoke toward the other participants. Then the Elder invites participants to smudge by fanning the smoke toward themselves (Schirch, 2005, p. 66). They scoop it over their heads and down their faces and bodies so that the smoke fills the air, and then end by taking a deep breath of it before the meeting or conference begins. Here, a set of actions, smells, and visual symbols creates a unique space that symbolically supports, and thereby scaffolds, the work of building peace. In part this expressive activity functions so as to evoke a particular mood and foster a particular sort of affective orientation among participants. Smells, shared movements, deep breathing, and the fanning of the smoke together create an affective niche that not only alters how the parties *feel*, but also changes their overall cognitive outlook and perspective. The smudging ritual helps individuals to focus on why they are there and what sorts of relationships they hope to develop (Schirch, 2005, p. 73), and this sort of reframing is central to building peace.

Like ritual, performance art sometimes plays a powerful role in bringing about constructive change. This sort of artistic expression involves emotional and sensual engagement and allows parties to tap into the relational and spiritual dimensions of conflict. As emotions undergo transformation, parties begin to frame and interpret their situation in new ways. For example, in the 1980s, when international mediation efforts to stop the war between Mali and Burkina Faso were failing, the president of neighboring Guinea persuaded the presidents of these two countries to attend a meeting at his palace. There, in front of the Presidential Palace, one of West Africa’s celebrated praise singers (Kanja Kouyate) performed for the visiting presidents. Through poetry, song, and dance, Kanja Kouyate called on the presidents to lead their people toward peace. He appealed to their human goodness as leaders and asked them to free their peoples from the shame and suffering of continued conflict. The performance was so emotional that the two presidents shed tears, embraced, and publicly vowed not to return to war. In this instance, artistic expressive helped to catalyze an *experiential break*, one which affectively “moved” the parties involved, disrupted their existing schemas, and ultimately shifted the way that they framed the conflict and envisioned ways forward. Within the next 2 months, they had signed a lasting peace agreement (Lederach, 2005, p. 155). It seems clear that this introduction of poetry, song, and dance functioned as crucial *affective-cognitive scaffolding*: Kouyate’s performance not only influenced the affective states of those watching, but also created an environment in which the leaders could envision a way forward. This is because Kouyate’s desires and perspective, as expressed through his singing, influenced the way in which those watching the performance made sense of themselves and their surroundings. By modulating their emotions, Kouyate’s singing was able to bring about a shift in perspective and point of view. This illustrates, once again, how affective scaffolds simultaneously buttress cognitive capacities, and also how affective reorientation often goes hand in hand with cognitive transformation.

I have conceptualized this formation of new interpretive frames and habits of attention in terms of affective framing. The basic idea is that by tapping into motivational, emotional, and relational elements of subjects’ lives, expressive activities modulate the affective frames of the parties involved. These new modes of affective attunement constitute a modification in perspective and an alteration in patterns of emotional sensitivity. I hypothesize that Kouyate’s performance altered the participants’ emotional state, allowing for a change in their affective frames and habits of bodily attunement, and that as a result of a shift in affective framing, participants actually became more *receptive* to certain kinds of information, more “open” to alternative points of view, and more able to appreciate the relevance and salience of factors that previously had remained obscure (e.g., both sides’ shared desire for peace). This suggests that affective reframing has the potential to change subjects’ sense of what is true or inevitable, help them to imagine alternative ways in which the world might be structured, and allow them to envision new modes of social interaction. It also may help subjects to become more keenly aware of pertinent facts, more capable of detecting oversimplifications, and more receptive and responsive to the concerns of others. What is more, it can dramatically change their sense of what sorts of actions are possible, opening up new avenues for social engagement and response.

## A Concluding Cautionary Note

I have argued that the evocation of emotion can lead to a shift in affective framing and thereby make a positive contribution to self-knowledge and interpersonal understanding. One might object, however, that because affectivity and emotion typically have a distorting influence on cognition, affective scaffolding may very well detract from subjects’ cognitive capacities rather than scaffolding them. We know that emotions sometimes ‘skew the epistemic landscape’ (Goldie, 2004, p. 99) by highlighting features of the world that *are not truly relevant*, steeping us in bias, or blinding us to certain aspects of our situation. Instances when affective framing leads to error include moments when we “actually only [look] at the world through the lens of our disgust, or our shame, and mistakenly [suppose] the evoking situation to have the kind of morally significant features that would warrant these emotions” (Jones, 2006, p. 48). Maladaptive habits of mind and emotional reactivity can serve to reinforce false assumptions, solidify unhealthy feelings, and render subjects unable to appraise things accurately and realistically. Once we understand emotion’s role in providing us with an interpretive lens, the danger of evoking emotions that lead to a warped outlook becomes readily apparent. It is true that the evocation of certain kinds of affective states sometimes distorts perception, obscure the truth, and contribute to faulty assumptions and interpretations. Intense emotions of grief, anger, or fear even can lead to “cognitive blindness”: people see what they feel, and they are so caught up in their own feelings and perspective that they become blind to alternative ways of viewing things. Thus, there is a danger that the use of expressive arts will evoke emotions that negatively impact the processes of therapy and peacebuilding.

It is important to acknowledge, then, that the use of affective scaffolds to evoke emotion is a risky endeavor and that expressive activity must be introduced thoughtfully and carefully so as to ensure that it makes a positive contribution to cognition. Especially at the beginning stages, great emphasis should be placed on fostering feelings of empathy and respect and building positive rapport among the participants. It also is important to provide a semi-structured format that can serve both as a safe space, as well as a setting for some degree of spontaneity and emotional expression. The fact that DMT often plays such a productive role in the therapeutic process suggests that it is possible to organize settings in such a way that they cultivate emotional expression and, simultaneously, help participants to be more accurate, effective, and insightful cognizers. There is little doubt, obviously, that critical reflection and reasoning likewise play a pivotal role in the cognitive processes associated with self-understanding and social cognition. With the help of rational assessment, subjects can shift their perspectives, alter their affective orientation, and gain new understandings. Just as cognitive processes are partly a matter of affective framing, the sort of affective framing patterns that a person develops are partly a matter of reflective thought processes. My central claim is simply that affective reframing can make a significant contribution to both therapy and peacebuilding, and that when it does, this serves as powerful illustration of the way in which affective scaffolds can serve, simultaneously, as scaffolding for cognition. More research should be done to investigate how expressive arts might be utilized as affective-cognitive scaffolding.

## Author Contribution

The author confirms being the sole contributor of this work and approved it for publication.

## Conflict of Interest Statement

The author declares that the research was conducted in the absence of any commercial or financial relationships that could be construed as a potential conflict of interest.
